# Insights into the respiratory tract microbiota of patients with cystic fibrosis during early *Pseudomonas aeruginosa* colonization

**DOI:** 10.1186/s40064-015-1207-0

**Published:** 2015-08-09

**Authors:** Marlène Keravec, Jérôme Mounier, Emmanuel Prestat, Sophie Vallet, Janet K Jansson, Gaëtan Burgaud, Sylvain Rosec, Stéphanie Gouriou, Gilles Rault, Emmanuel Coton, Georges Barbier, Geneviève Héry-Arnaud

**Affiliations:** EA 3882-Laboratoire Universitaire de Biodiversité et Ecologie Microbienne (LUBEM), Groupe de Bactériologie-Virologie, Faculté de Médecine et des Sciences de la Santé de Brest, University of Brest, 22 Avenue Camille Desmoulins, C.S. 93837, 29238 Brest Cedex 3, France; Earth Sciences Division, Lawrence Berkeley National Laboratory, Berkeley, CA USA; Department of Bacteriology-Virology, Hygiene and Parasitology-Mycology, University Hospital of Brest, Brest, France; Biological Sciences Division, Pacific Northwest National Laboratory, Richland, USA; INSERM CIC 0502, University Hospital of Brest, Brest, France; CF Center, Roscoff, France; QIAGEN Marseille SA, Research & Development Luminy Biotech Enterprises, Marseille, France

**Keywords:** Cystic fibrosis, Respiratory tract microbiota, *Pseudomonas aeruginosa*, Early colonization, Anaerobes, Respiratory viruses

## Abstract

*Pseudomonasaeruginosa* plays a major role in cystic fibrosis (CF) progression. Therefore, it is important to understand the initial steps of *P. aeruginosa* infection. The structure and dynamics of CF respiratory tract microbial communities during the early stages of *P. aeruginosa* colonization were characterized by pyrosequencing and cloning-sequencing. The respiratory microbiota showed high diversity, related to the young age of the CF cohort (mean age 10 years). Wide inter- and intra-individual variations were revealed. A common core microbiota of 5 phyla and 13 predominant genera was found, the majority of which were obligate anaerobes. A few genera were significantly more prevalent in patients never infected by *P. aeruginosa.* Persistence of an anaerobic core microbiota regardless of *P. aeruginosa* status suggests a major role of certain anaerobes in the pathophysiology of lung infections in CF. Some genera may be potential biomarkers of pulmonary infection state.

**Electronic supplementary material:**

The online version of this article (doi:10.1186/s40064-015-1207-0) contains supplementary material, which is available to authorized users.

## Background

Chronic lung infections are the primary cause of morbidity and mortality in cystic fibrosis (CF) (Nixon et al. 2001). The respiratory tract of CF patients is colonized and infected by numerous bacteria from an early age. Among cultivable pathogens, which include *Pseudomonas aeruginosa*, *Staphylococcus aureus*, *Haemophilus influenzae*, *Burkholderia cepacia* complex and *Achromobacter xylosoxidans*, *P. aeruginosa* is the most prevalent pathogen in CF. In children with CF, Kosorok et al. (2001) demonstrated a longitudinal correlation between *P. aeruginosa* acquisition and gradual deterioration of pulmonary function. Therefore, early detection of *P. aeruginosa* appears crucial for maximizing the chances of efficiently controlling this pathogen, notably by early institution of anti-*Pseudomonas* antibiotherapy (Valerius et al. 1991).

Better understanding of the initial steps of *P. aeruginosa* infection would also help in preventing early colonization in CF airways. According to Klepac-Ceraj et al. (2010), the community composition of the CF pulmonary microbiota is a better indicator of disease progression than the presence of *P. aeruginosa* alone. The endogenous respiratory microbiota may modulate pathogenesis in a polymicrobial context through microbe–microbe and polymicrobe–host interactions (Sibley and Surette 2011). Several studies reported important differences in the structure of the respiratory microbiota between healthy subjects and patients with chronic lung diseases (Huang et al. 2010). The lung infections of CF patients are also considered as polymicrobial (Sibley and Surette 2011; Sibley et al. 2006). Recently, van der Gast et al. (2011) identified a core group dominated by *P. aeruginosa* and a satellite group composed of multiple microbial species, including species not routinely identified by culture and not recognized as CF pathogens. The inability of conventional infection models to detect a pathogenic response to certain microorganisms known as commensals should not mean that their potential to contribute to polymicrobial infection can be disregarded (Sibley et al. 2008). The CF lung infection model has thus moved from a classical to an ecological paradigm.

In the light of these new findings, the present study investigated whether early *P. aeruginosa* colonization in CF patients was accompanied by significant changes in respiratory microbiota in terms of community structure and relative abundance, with the challenge of identifying potential biomarkers or predictive factors of *P. aeruginosa* implantation.

## Methods

### Population and sampling

Five CF patients with an age range of 5–19 years were followed up during a median of 22 months [16–39 months]. Sputum samples were processed using the standard operating procedure (SOP) of the French guidelines (Anonyme 2010). Twenty spontaneous sputum samples (4 per patient) were collected and homogenized with an equal volume of dithiothreitol (Digest-EUR^®^ Eurobio, Courtabœuf, France) for 30 min at 37 °C. Clinical, therapeutic and biological data were compiled such as CFTR mutation, clinical state, antibiotic treatment and *P. aeruginosa* status. Sputum sample quality was verified by cytological examination of fresh smears and classified according to the number of epithelial cells and leukocytes. Sputum samples were classified in three quality classes: poor quality when the number of epithelial cells (cells/field) was ≥25 and the number of leukocytes (cells/field) was ≤10; appropriate quality when the number of epithelial cells was ≤25 and the number of leukocytes (cells/field) was ≥10. The other combinations between epithelial cells and leukocytes were considered of moderate quality. All samples were stored at −80 °C prior to DNA extraction. According to the Lee’s definition of *P. aeruginosa* infection status (Lee et al. 2003), three patients were categorized as ‘free’ (no culture of *P. aeruginosa* for at least the previous year), and two patients as ‘never’ (*P. aeruginosa* had never been detected from sputum or cough swabs culture). All patients became *P. aeruginosa* positive in culture during the follow-up.

### DNA extraction and quantitative PCR

Presence of *P. aeruginosa* was also investigated by quantitative PCR (qPCR) as previously described (Le Gall et al. 2013) (Additional file 1: Table S1). Bacterial DNA was extracted using the QIAamp DNA Mini Kit (QIAGEN, Courtabœuf, France) according to the manufacturer’s instructions; a sonication step of 5 min was applied prior to proteinase K digestion, which was performed for 3 h at 56 °C. The total bacterial load was established by qPCR with universal primers targeting the 16S rRNA gene as previously described (Zemanick et al. 2013).

### Barcoded pyrosequencing and bioinformatic analyses

The V3 and V4 hypervariable regions of the 16S rRNA gene were amplified using primers 347F (5′-GGAGGCAGCAGTRRGGAAT-3′) and 803R (5′-CTACCRGGGTATCTAATCC-3′) (Nossa et al. 2010). The 50 µl PCR mixture contained 10 pmol of each primer, 1× of polymerase buffer, 0.4 mM of each dNTP, 1.25 U of GoTaq^®^ Flexi DNA polymerase (Promega, France), 3 mM of MgCl_2_ and 50 ng of DNA template. Reactions were heated at 94 °C for 5 min followed by 30 cycles of 94 °C for 1 min, 58 °C for 40 s and 72 °C for 40 s, and with a final extension step of 5 min at 72 °C. Two independent PCR amplifications were carried out for each sputum sample and the resulting PCR products were pooled. Prior to pyrosequencing, the size and quantity of pooled amplicon libraries were determined by an Agilent 2100 Bioanalyzer (Agilent Technologies, Germany) and PCR products were sequenced on a Genome Sequencer FLX™ Titanium (454 Life Sciences Corp., Bradford, CT, USA) by GATC Biotech (Konstanz, Germany). Quality control and sequence processing were performed using the UPARSE pipeline (Edgar 2013).

Data set was processed and analyzed using the UPARSE pipeline with scripts available on drive5 (http://drive5.com). The following quality filtering parameters were applied: truncation length of 250 bp, truncation to the first nucleotide with a quality score under 20, maximal expected error of 0.25. After removal of singletons, sequences were clustered into OTUs based on a sequence similarity level of 0.97 using the UPARSE-OTU algorithm followed by filtration of chimeras against the ‘Gold’ database using UCHIME (Edgar 2013; Edgar et al. 2011).

Sequence data and the OTU table obtained using the UPARSE pipeline were then processed and analyzed using Quantitative Insights Into Microbial Ecology (QIIME) (Caporaso et al. 2010). The representative sequence of each OTU was classified in QIIME against the Greengenes database (http://greengenes.lbl.gov/, version released on May 2013) using the Ribosomal Database Project (RDP) classifier with a confidence threshold of 80 %. OTUs of interest were also further classified to the species level using the RDP SeqMatch tool. Prior to alpha and beta diversity analyses, the OTU table was rarefied to the smallest number of reads obtained in a sample. Alpha diversity was determined using the Shannon index, Chao-1 estimator and equitability (evenness) index. Beta diversity was assessed using the Adonis test and the Bray–Curtis distance and visualized by principal coordinate analysis (PCoA). Diversity was also evaluated using the Simpson’s diversity index (SDI) transformed with the arcsine square root. A non-parametric (Kruskal–Wallis test) statistical test was applied in QIIME to test whether genera abundance was significantly associated with their associated metadata. A hierarchical ascendant classification (HAC) by Euclidean distance and an abundance heatmap were also obtained using XLstat software package (http://www.xlstat.com). The core microbiota was defined as OTUs present in at least 50 % of samples at a minimum relative abundance of 0.1 % of the total bacterial community. Sequences of the selected OTUs were aligned to the Greengenes core-set-aligned using PyNast with default parameters. The concordance between detection of *P. aeruginosa* using 454 pyrosequencing and culture based-method was assessed. The alignment was then filtered to remove gaps and hypervariable regions using a lane mask and a tree was generated using FastTree (Price et al. 2009). A circular phylogenetic tree was then constructed using the Interactive Tree Of Life (iTOL) (Letunic and Bork 2007). The sequence data were deposited at the NCBI Short Read Archive (BioProject no. PRJNA258440).

### Respiratory viruses screening

One sample (A1) was not included in this screening as having not enough DNA. From the remaining 19 samples, viral RNA and DNA were extracted using the automated NUCLISENS^®^ easyMAG™ (bioMérieux, Marcy l’Etoile, France) after a treatment with 25 µl of proteinase K (10 mg/ml) during 2 h at 56 °C. The nucleic acids were eluted in 50 µl and conserved at −80 °C. The RespiFinder^®^ SMART 22 FAST (PathoFinder, Maastricht, The Netherlands) was used according to the manufacturer’s instructions and then, qPCR was performed in a GeneAmp^®^ PCR System 9700 (Applied Biosystems, Courtabœuf, France). The kit simultaneously detects 18 respiratory viruses (Influenza A, Influenza B, Influenza A H1N1v, Respiratory Syncytial Virus A, Respiratory Syncytial Virus B, Parainfluenza 1, Parainfluenza 2, Parainfluenza 3, Parainfluenza 4, Coronavirus OC43, Coronavirus 229E, Coronavirus NL63, Coronavirus HKU1, Rhinovirus/enterovirus, Adenovirus, human Metapneumovirus, Bocavirus Type 1) and four bacteria (*Chlamydophila pneumoniae*, *Mycoplasma pneumoniae*, *Legionella pneumophila*, *Bordetella pertussis*).

### Cloning-sequencing analysis

For the cloning and sequencing analysis, the near-full-length 16S rRNA gene was amplified using universal primers pA (5′-AGAGTTTGATCCTGGCTCAG-3′) and pH (5′-AAGGAGGTGATCCAGCCGCA-3′) (Turner et al. 1999). PCR amplification was performed in a total volume of 25 µl containing 2.5 pmol of each primer, 1× polymerase buffer, 0.2 mM of each dNTP, 0.625 U GoTaq polymerase and 2 mM MgCl_2_ and 50 mg DNA template. Reaction was heated at 94 °C for 5 min followed by 30 cycles of 94 °C for 60 s, 57 °C for 40 s and 72 °C for 60 s followed by a final extension at 72 °C for 10 min. After PCR, the amplification products approx. 1,500 bp in size were checked on 1 % agarose gel (Promega, France). The PCR products obtained were ligated in pCR4-TOPO vector (Invitrogen, Carlsbad, CA, USA). The recombinant plasmids were used to transform *Escherichia coli* strain TOP 10 One Shot chemically competent cells according to the manufacturer’s instructions. Restriction fragment length polymorphism (RFLP) was used for dereplication of clone libraries as described previously (Mounier et al. 2009) using *Hae*III restriction enzyme. Clone representatives of each clone library showing distinct restriction profiles were then sequenced at the Biogenouest sequencing platform in the *Station Biologique de Roscoff* center (http://www.sb-roscoff.fr). The sequences were assembled into contigs using DNA Baser software (http://www.dnabaser.com) and compared with the NCBI database (http://www.ncbi.nlm.nih.gov/BLAST), Greengenes (http://greengenes.lbl.gov/, version released on May 2013) and RDP SeqMatch tool program to obtain S_ab values with database sequences. Clone sequences and sequences with the highest S_ab score retrieved from GenBank were then aligned using ClustalW. The coverage percentage of clone library coverage using the Good’s formula and alpha diversity were calculated.

## Results and discussion

### Diversity of the respiratory microbiota

Out of 625,263 reads generated using pyrosequencing, 287,306 high-quality reads were retained, with a mean 14,365 reads per sample (7,954–29,458) (Additional file 2: Table S2). After normalization to the sample with the lowest number of reads (7,954), 159,060 reads (mean length approx. 420 bp) were analyzed; 124 OTUs were identified (32–85 per sample). Three samples (C2, D3 and D4) were not included in clone libraries because small numbers of clones were obtained for these samples. From the remaining 17 samples, 1,327 clones were analyzed, representing 35 OTUs. According to Good’s formula, the coverage of clone libraries ranged from 77 to 99 %, indicating that the most abundant taxa were represented in the libraries.

Bacterial diversity was high, with a mean Shannon index of 3.58 (1.20–4.50) which was in accordance with others studies (Boutin et al. 2015; Bernarde et al. 2015) (Additional file 2: Table S2). The majority of bacteria were represented (Good’s coverage of 0.999). Moreover, the Chao1 richness index was only slightly higher than the number of observed OTUs in each sample, indicating that true bacterial richness was not underestimated. An Adonis test was performed and no significant impact of the cytological score was observed (*p* > 0.1, R^2^ = 0.19). The Bray Curtis dissimilarity was evaluated between successive samples (Additional file 3: Table S3) and showed some degree of fluctuations. The SDI values were calculated to assess bacterial diversity and showed increasing trends across time (y = 0.0496x + 0.8775, R^2^ = 0.7314). The high diversity found in the present study can be explained by the young age of these patients (mean age 10 years) because microbial diversity is maximal in this age range (Cox et al. 2010). However, such age correlation is still an open question and further studies are needed to confirm these findings.

### Core CF pulmonary microbiota in children is characterized by ‘oral’ anaerobic bacteria

**Fig. 1:**
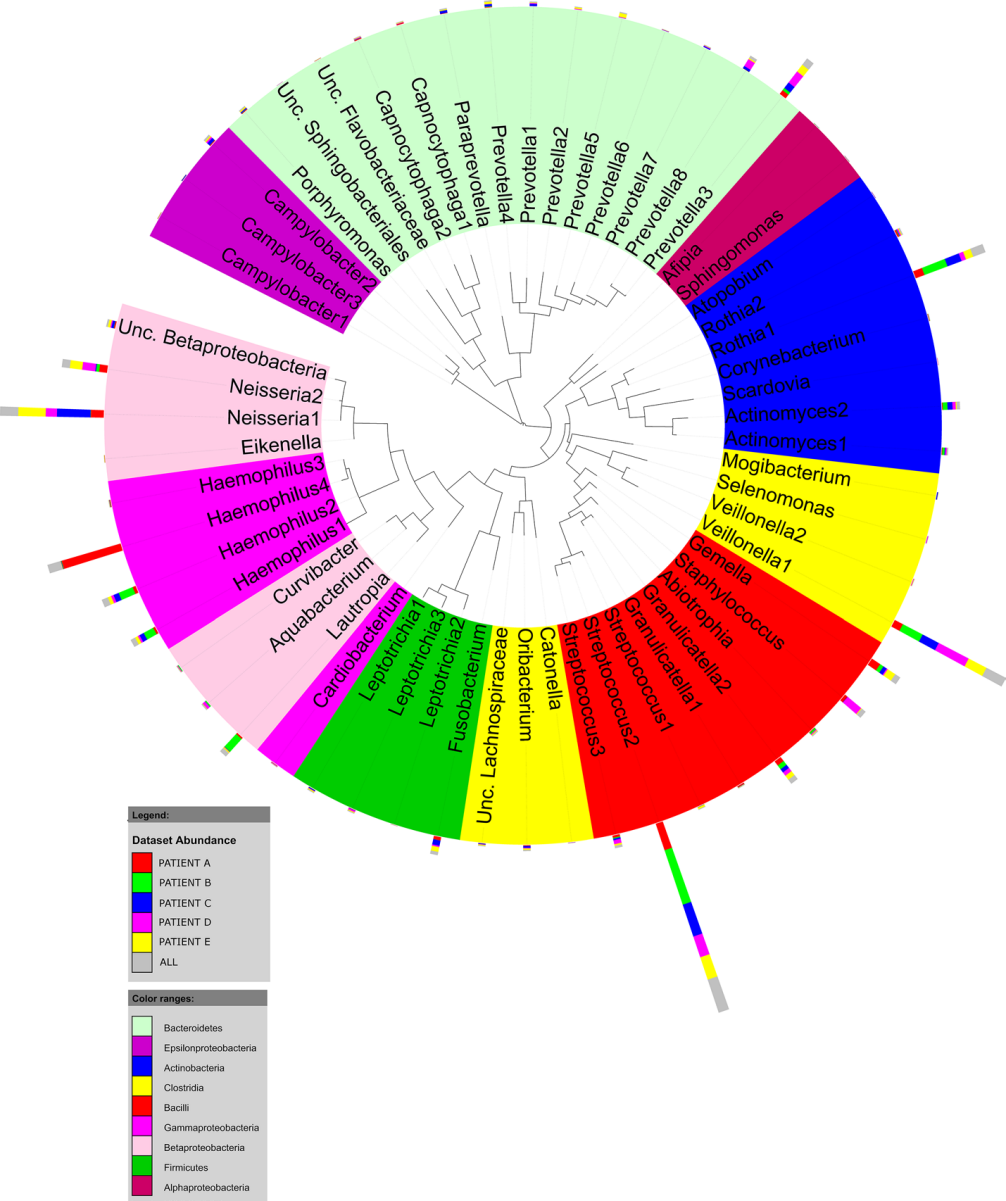
Phylogenetic tree of the bacterial diversity of core CF pulmonary microbiota. This figure was created using the interactive Tree Of Life (iTOL) (Letunic and Bork 2007). The *inner circle* shows genera colored by phylum or *Proteobacteria* class. *Each node* represents a core OTU shared among 50 % of the samples at a relative abundance >0.1 % of the total bacterial community. The *outer bars* represent the relative abundance of each OTU for the 5 CF patients.

Fifty-seven OTUs formed the core microbiota (Additional file 4: Fig. S1a). As shown in Fig. 1 and Additional file 4: Fig. S1b, five phyla were identified: *Firmicutes*, *Proteobacteria*, *Actinobacteria*, *Bacteroidetes* and *Fusobacteria*, and were also detected by cloning-sequencing in similar proportions. The core CF pulmonary microbiome was composed of 13 predominant genera (relative abundance >1 %) whose sum equaled 94 % of the ‘core’ reads (Additional file 5: Fig. S2). Eight of these 13 genera were also retrieved by cloning-sequencing, which in turn did not retrieve genera not found using 16S rRNA pyrosequencing. No remarkable differences were observed between the two techniques in terms of relative abundance at phylum or genus level. The detection of *P. aeruginosa* using 454 pyrosequencing was in good agreement with that using qPCR and culture based-method. Indeed, reads of *P. aeruginosa* were obtained in all but one *P. aeruginosa* positive-sample from which *P. aeruginosa* was only detected using qPCR.

The 13 predominant genera identified in the present study are commonly found in CF respiratory tract microbiota (Willner et al. 2012; Venkataraman et al. 2015; Tunney et al. 2008; Carmody et al. 2013). Nine of them have been described to be the most abundant genera in healthy lungs (Wat et al. 2008). In agreement with previous studies, there was a large abundance of obligate anaerobes such as *Veillonella* and *Prevotella* (Tunney et al. 2008; Cheung et al. 2013), and facultative anaerobes such as *Gemella,* which is thought to be a biomarker for the exacerbation in CF lung (Carmody et al. 2013). OTUs corresponding to uncultivable bacteria affiliated to the TM6, TM7 and WPS-2 phyla also reported to be components of the oral microbiota (Adler et al. 2013), were present at low abundances in the sputum samples. While TM7 has already been detected in CF sputum samples (Blainey et al. 2012), this is the first time, to our knowledge, that TM6 and WPS-2 are reported in CF. The large abundance of so-called ‘oral bacteria’ in the respiratory microbiota of CF children corroborates the viewpoint that the oral cavity is a potential source of pathogens and other bacteria such as anaerobes that reach and colonize the lower airways of CF patients (Boutin et al. 2015; Rivas Caldas and Boisramé 2015). In fact, undoubtedly, upper and lower airways are interconnected and it would be interesting to determine the origin of pathogens to better understand their colonization process in CF.

### High viral prevalence in CF sputum samples

In 52.6 % of samples (n = 10), at least one respiratory virus was detected while ≥2 viruses were found in 15.8 % of samples (n = 3). The most frequent viruses detected were human picornaviruses (rhinovirus or enterovirus) with a prevalence of 36.8 % (n = 7) in 4 of the 5 CF patients (Additional file 1: Table S1). We also detected in low proportion (n = 1) bocavirus type 1, parainfluenzae type 1 and type 2, coronavirus NL63, and influenzae A/H1N1v. There was no link between virus prevalence and *P. aeruginosa* abundance (Kruskal–Wallis test, FDR corrected >0.5). Rhinoviruses are commonly detected in CF children and are frequently associated with pulmonary exacerbations with worse severity in young CF patients (Asner et al. 2012). In accordance with the findings of Asner et al. (2012), peak prevalence of rhinovirus occurs in spring and fall months. It has been hypothesized that respiratory viruses could improve the acquisition of bacterial pathogens (Wat et al. 2008). In further study, it would be interesting to determine the influence of respiratory viruses on CF pulmonary microbiota.

### CF respiratory tract microbiota dynamics throughout *P. aeruginosa* early colonization

#### Stability of the microbial structure in the early stages of *P. aeruginosa* colonization

**Fig. 2:**
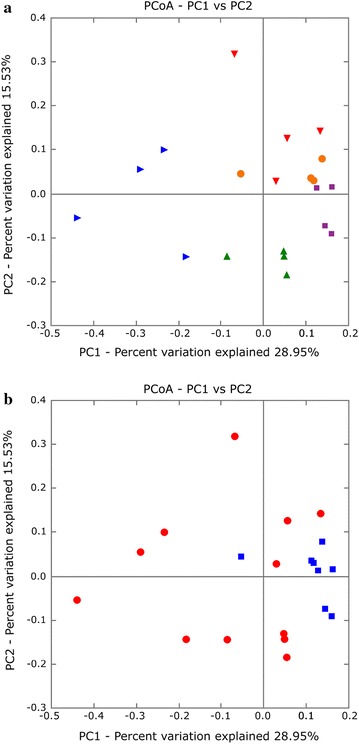
PCoA of community structures using Bray–Curtis distances. **a** Community structures from each patient (patient A, *red inverted triangles*; patient B, *blue triangles*; patient C, *orange circles*; patient D, *green triangles*; patient E, *purple squares*) and **b** of samples from ‘Never’ (*red circles*) and ‘Free’ patients (*blue squares*).

As depicted in Fig. 2a, sample grouping by patient was statistically significant (Adonis: *p* < 0.001, R^2^ = 0.52). The natural propensity of the lung microbiome to diverge between individuals strengthens the choice of a study design based on longitudinal follow-up of a few CF patients rather than a cross-sectional study of a large number of patients. The mean quantity of 16S rRNA gene copies/ml was 7.39 log_10_ ± 0.49. Fluctuations [6.28 log_10_ − 7.98 log_10_] were observed in biomass abundance but without any impact on microbial community structure (Adonis: *p* = 0.3946, R^2^ = 0.054), which is in contrast with a previous study suggesting a positive correlation between an increased microbial colonization and a decreased microbial diversity (Boutin et al. 2015). Those differences might be explained by the difference in the microbiological features of the CF cohort as part of the cohort explored by Boutin et al. (2015) was chronically colonized by *P. aeruginosa*. These data underlined that, besides age, it is crucial to well define the *P. aeruginosa* colonization’s status at time of sampling following Lee’s criteria (Lee et al. 2003).

#### Persistence of anaerobes

**Fig. 3:**
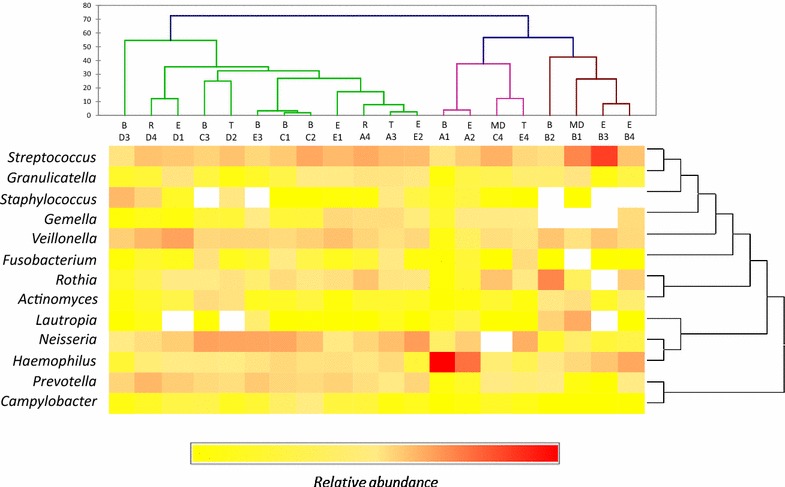
Cluster analysis of bacterial communities of CF sputa and relative abundance of the predominant genera shared between 50 % of samples and accounting for >1 % of total bacterial community. Hierarchical ascendant classification (HAC) of the 13 predominant genera found in sputum samples by Euclidean distance. Each clinical BETR stage is indicated (baseline clinical state, B; pulmonary exacerbation, E; treatment for exacerbation, T; recovery, R; missing data, MD). The relative abundance for each genus is colored in *shades* of *red* (high relative abundance) to *yellow* or *bright white* (low relative abundance), as shown in the *color* key.

Along with the early *P. aeruginosa* colonization process, nine OTUs were considered persistent i.e., present at high relative abundance in all samples. They comprised six genera, including three obligate anaerobes (*Veillonella*, *Actinomyces* and *Prevotella*), and three facultative anaerobes (*Haemophilus*, *Granulicatella* and *Streptococcus*) (Fig. 3) not presently assessed on SOP, except for *Haemophilus* spp. The genera *Streptococcus* comprised *S. mitis*, *S. anginosus* (formerly called *S. milleri* group) and *S. salivarius* groups. Anaerobes undoubtedly play a major role in the pathophysiology in CF patients because they are involved in inflammation, infection and lung function (Tunney et al. 2008; O’Neill et al. 2015). In the present study, the issue was to know whether *P. aeruginosa* implantation was subsequent to a proliferation of anaerobes, or the contrary (chicken-and-egg problem). As the same anaerobic genera were detected in patients initially classified as ‘never’, which became *P. aeruginosa*-positive a few months later, this might suggest that anaerobes do not impede implantation of *P. aeruginosa*. Indeed, mass-DNA sequencing revealed that, besides the well-known lung pathogens, a core microbiota including four persistent anaerobes genera (*Veillonella*, *Streptococcus*, *Actinomyces* and *Prevotella*) exists and is common to pulmonary diseases (Cheung et al. 2013). Rogers et al. (2015) recently described the ‘like begets like’ phenomenon, whereby most dominant pathogens in microbiota are determinant for the implantation of *P. aeruginosa.* The role of these four genera as potential enhancers of *P. aeruginosa* colonization remains to be clearly demonstrated.

### A specific microbiota composition relative to Lee’s status?

A way to evaluate whether *P.aeruginosa* had impacted the airway microbiota was to compare ‘never’ and ‘free’ patients’ microbiota. The difference in bacterial density and in the number of reads of *P. aeruginosa* between ‘never’ and ‘free’ patients was not statistically significant (*p* > 0.05) (Additional file 1: Table S1). Thus, bacterial density did not appear to be a potential marker of *P. aeruginosa* implantation. Similarly, Shannon’s index and equitability were not significantly different between ‘free’ and ‘never’ CF patients. Conversely, richness was significantly greater (*p* < 0.05) in ‘never’ [Chao-1: 79.8 (72.03–87.61); observed species: 71.6 (63.85–79.43)] than in ‘free’ patients [Chao-1: 62.7 (52–73.5); observed species: 55.9 (45.24–66.71)] (Additional file 2: Table S2). Moreover, as depicted in Fig. 2b, the structure of the microbiota was affected by the Lee’s status (see also Additional file 6: Fig. S3). Indeed, a significant clustering by Lee’s status was observed (Adonis test p = 0.0199, R^2^ = 0.105). The present results may suggest that the richness of the CF respiratory microbiota is directly negatively impacted by first acquisition of *P. aeruginosa*; this very preliminary data has to be reinforced with a larger sample dataset.

## Conclusions

The present study allowed identifying bacterial species, including anaerobic bacteria, the role of which may be underestimated and which may be indirectly implicated in *P. aeruginosa* lung infection pathogenesis. In addition, this study demonstrated the impact of *P. aeruginosa* status on the composition of the CF lung microbiota. It was confirmed that core OTUs including anaerobes, were a common denominator in CF patients.

Further studies are needed to confirm these results and thereby improve our knowledge of the early stages of *P. aeruginosa* colonization. This pilot study underlines the importance of characterizing microbial communities associated with pulmonary clinical profiles, as longitudinal follow-up of CF patients could improve our overall view of the pathophysiology of lung infection in CF patients. Furthermore, the application of strategies such as respiratory microbiota transplantation or probiotics could delay pathogen implantation in CF patients. Henceforth, is it clearly demonstrated the crucial importance to follow the evolution of the pulmonary microbiota of CF patients allowing to monitor the efficiency of treatments and determine valuable predictive biomarkers.

## Additional files

Additional file 1: Table S1. Clinical data and detection of *P. aeruginosa* in CF sputum samples. Additional file 2: Table S2. Pyrosequencing parameters and alpha diversity. Additional file 3: Table S3. RDP taxonomic assignment for each OTU found with relative abundance >1%. Additional file 4: Fig. S1. a - Number of core OTUs shared between samples at different prevalence thresholds. Only 9 OTUs were shared between all samples. Fig. S1b - Relative abundance of the most abundant taxa composing the common core microbiota (found across 50% of samples) present in the lungs of CF patients. Five phyla were found: *Actinobacteria* (10.4%), *Bacteroidetes* (9.6%), *Firmicutes* (43.7%), *Fusobacteria* (2.5%) and *Proteobacteria* (33.9%), and 13 predominant (i.e., relative abundance > 1%) genera: *Haemophilus* (14%), *Campylobacter* (1%), *Neisseria* (14.5%), *Lautropia* (2.4%), *Fusobacterium* (1.8%), *Veillonella* (12.3%), *Staphylococcus aureus* (2.6%), *Streptococcus* (2.1%), *Granulicatella* (2.9%), *Gemella* (3.3%), *Prevotella* (8.0%), *Rothia* (7.8%) and *Actinomyces* (2.4%). Additional file 5: Fig. S2. Bacterial community composition of serial samples obtained from 5 CF patients and quantification of total bacterial density. Relative abundance of each genus accounting for >1 % of the total bacterial community is shown and the relative abundance of other genera (accounting for <1 %) is shown in gray. Circles indicate total bacterial density (16S rRNA copies/mL sputum) based on quantitative PCR. Black stars and red stars indicate sputum samples positive for *P. aeruginosa* by culture and 454 pyrosequencing, respectively. Additional file 6: Fig. S3. Taxa summary at the genus level of the CF pulmonary microbiota.

## References

[CR1] Adler CJ, Dobney K, Weyrich LS, Kaidonis J, Walker AW, Haak W (2013). Sequencing ancient calcified dental plaque shows changes in oral microbiota with dietary shifts of the Neolithic and Industrial revolutions. Nat Genet.

[CR2] Anonyme (2010) Recommandations pour l’analyse bactériologique des prélèvements d’expectoration chez les patients atteints de mucoviscidose. In: REMIC: Référentiel en Microbiologie Médicale (ed) Société Française de Microbiologie, pp 99–104

[CR3] Asner S, Waters V, Solomon M, Yau Y, Richardson SE, Grasemann H (2012). Role of respiratory viruses in pulmonary exacerbations in children with cystic fibrosis. J Cyst Fibros.

[CR4] Bernarde C, Keravec M, Mounier J, Gouriou S, Rault G, Férec C (2015). Impact of the CFTR-potentiator ivacaftor on airway microbiota in cystic fibrosis patients carrying a G551D mutation. PLoS One.

[CR5] Blainey PC, Milla CE, Cornfield DN, Quake SR (2012). Quantitative analysis of the human airway microbial ecology reveals a pervasive signature for cystic fibrosis. Sci Transl Med.

[CR6] Boutin S, Graeber SY, Weitnauer M, Panitz J, Stahl M, Clausznitzer D (2015). Comparison of microbiomes from different niches of upper and lower airways in children and adolescents with cystic fibrosis. PLoS One.

[CR7] Caporaso JG, Kuczynski J, Stombaugh J, Bittinger K, Bushman FD, Costello EK (2010). QIIME allows analysis of high-throughput community sequencing data. Nat Methods.

[CR8] Carmody LA, Zaho J, Schloss PD, Petrosino JF, Murray S, Young VB (2013). Changes in cystic fibrosis airway microbiota at pulmonary exacerbation. Am Ann Thorac Soc.

[CR9] Cheung MK, Lam WY, Fung WYW, Law PTW, Au CH, Nong W (2013). Sputum microbiota in tuberculosis as revealed by 16S rRNA pyrosequencing. PLoS One.

[CR10] Cox MJ, Allgaier M, Taylor B, Baek MS, Huang YJ, Daly RA (2010). Airway microbiota and pathogen abundance in age-stratified cystic fibrosis patients. PLoS One.

[CR11] Edgar RC (2013). UPARSE: highly accurate OTU sequences from microbial amplicon reads. Nat Methods.

[CR12] Edgar RC, Haas BJ, Clemente JC, Quince C, Knight R (2011). UCHIME improves sensitivity and speed of chimera detection. Bioinformatics.

[CR13] Huang YJ, Nelson CE, Brodie EL, DeSantis TZ, Baek MS, Liu J (2010). Airway microbiota and bronchial hyper responsiveness in patients with sub-optimally controlled asthma. J Allergy Clin Immunol.

[CR14] Klepac-Ceraj V, Lemon KP, Martin TR, Allgaier M, Kembel SW, Knapp AA (2010). Relationship between cystic fibrosis respiratory tract bacterial communities and age, genotype, antibiotics and *Pseudomonas aeruginosa*. Environ Microbiol.

[CR15] Kosorok MR, Zeng L, West SHE, Rock MJ, Splaingard ML, Laxova A (2001). Acceleration of lung disease in children with cystic fibrosis after *Pseudomonas aeruginosa* acquisition. Pediatr Pulmonol.

[CR16] Le Gall F, Le Berre R, Rosec S, Hardy J, Gouriou S, Boisramé-Gastrin S (2013). Proposal of a quantitative PCR-based protocol for an optimal *Pseudomonas aeruginosa* detection in patients with cystic fibrosis. BMC Microbiol.

[CR17] Lee TWR, Brownlee KG, Conway SP, Denton M, Littlewood JM (2003). Evaluation of a new definition for chronic *Pseudomonas aeruginosa* infection in cystic fibrosis patients. J Cyst Fibros.

[CR18] Letunic I, Bork P (2007). Interactive Tree Of Life (iTOL): an online tool for phylogenetic tree display and annotation. Bioinformatics.

[CR19] Mounier J, Monnet C, Jacques N, Antoinette A, Irlinger F (2009). Assessment of the microbial diversity at the surface of Livarot cheese using culture-dependent and independent approaches. Int J Food Microbiol.

[CR20] Nixon GM, Armstrong DS, Carzino R, Carlin JB, Olinsky A, Robertson CF (2001). Clinical outcome after early *Pseudomonas aeruginosa* infection in cystic fibrosis. J Pediatr.

[CR21] Nossa CW, Oberdorf WE, Yang L, Aas JA, Paster BJ, DeSantis TZ (2010). Design of 16S rRNA gene primers for 454 pyrosequencing of the human foregut microbiome. World J Gastroenterol.

[CR22] O’Neill K, Bradley JM, Johnston E, McGrath S, McIlreavey L, Rowan S (2015). Reduced bacterial colony count of anaerobic bacteria is associated with a worsening in lung clearance index and inflammation in cystic fibrosis. PLoS One.

[CR23] Price MN, Dehal PS, Arkin AP (2009). FastTree: computing large minimum evolution trees with profiles instead of a distance matrix. Mol Biol Evol.

[CR24] Rivas Caldas R, Boisramé S (2015). Upper aero-digestive contamination by *Pseudomonas aeruginosa* and implications in cystic fibrosis. J Cyst Fibros.

[CR25] Rogers GB, van der Gast C, Serisier DJ (2015). Predominant pathogen competition and core microbiota divergence in chronic airway infection. ISME J.

[CR26] Sibley CD, Surette MG (2011). The polymicrobial nature of airway infections in cystic fibrosis. Cangene Gold Medal Lecture. Can J Microbiol.

[CR27] Sibley CD, Rabin H, Surette MG (2006). CF: a polymicrobial infectious disease. Future Microbiol.

[CR28] Sibley CD, Duan K, Fischer C, Parkins MD, Storey DG, Rabin HR (2008). Discerning the complexity of community interactions using a Drosophila model of polymicrobial infections. PLoS Pathog.

[CR29] Tunney MM, Field TR, Moriarty TF, Patrick S, Doering G, Muhlebach MS (2008). Detection of anaerobic bacteria in high numbers in sputum from patients with cystic fibrosis. Am J Respir Crit Care Med.

[CR30] Turner S, Pryer KM, Mia VPW, Palme JD (1999). Investigating deep phylogenetic relationships among cyanobacteria plastids by small subunit rRNA sequence analysis. J Eukaryot Microbiol.

[CR31] Valerius NH, Koch C, HØiby N (1991). Prevention of chronic *Pseudomonas aeruginosa* colonisation in cystic fibrosis by early treatment. Lancet.

[CR32] van der Gast CJ, Walker AW, Stressmann FA, Rogers GB, Scott P, Daniels TW (2011). Partitioning core and satellite taxa from within cystic fibrosis lung bacterial communities. ISME J.

[CR33] Venkataraman A, Bassis CM, Beck JM, Young VB, Curtis JL, Huffnagle GB (2015). Application of neutral community model to assess structuring of the human lung microbiome. mBio.

[CR34] Wat D, Gelder C, Hibbitts S, Cafferty F, Bowler I, Pierrepoint M (2008). The role of respiratory viruses in cystic fibrosis. J Cyst Fibros.

[CR35] Willner D, Haynes MR, Furlan M, Schmieder R, Lim YW, Rainey PB (2012). Spatial distribution of microbial communities in the cystic fibrosis lung. ISME J.

[CR36] Zemanick HJK, Wagner BD, Robertson CE, Sagel SD, Stevens MJ, Accurso FJ (2013). Inflammation and airway microbiota during cystic fibrosis pulmonary exacerbations. PLoS One.

