# Supplementary figures and images for: Insights into the respiratory tract microbiota of patients with cystic fibrosis during early Pseudomonas aeruginosa colonization
Source: Springerplus. 2015 Aug 9;4:405. doi: 10.1186/s40064-015-1207-0 (PMC4529844; doi:10.1186/s40064-015-1207-0)

a)

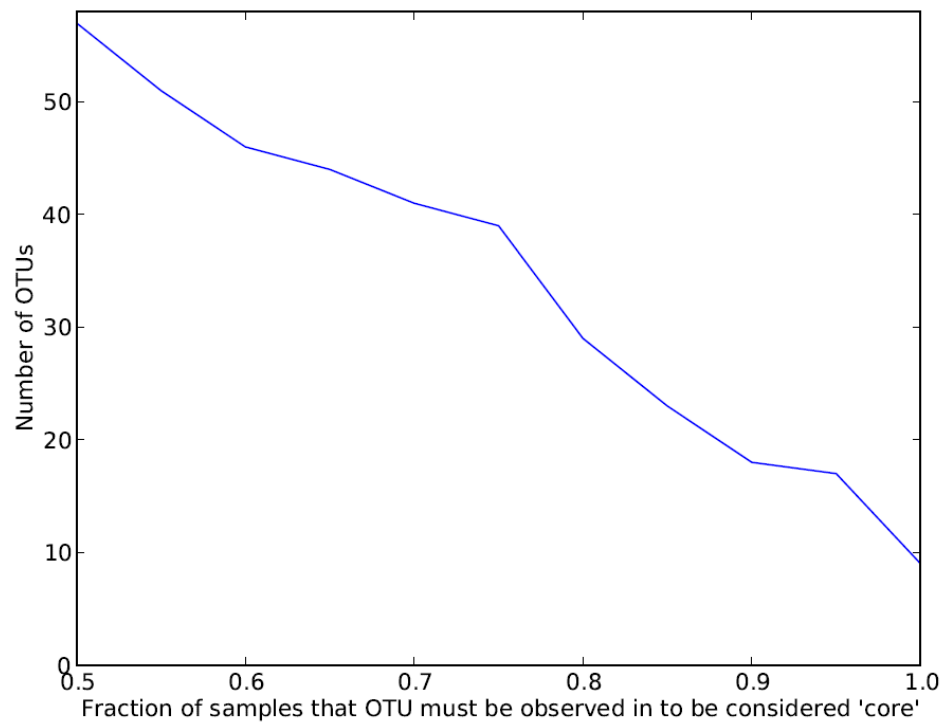

b)

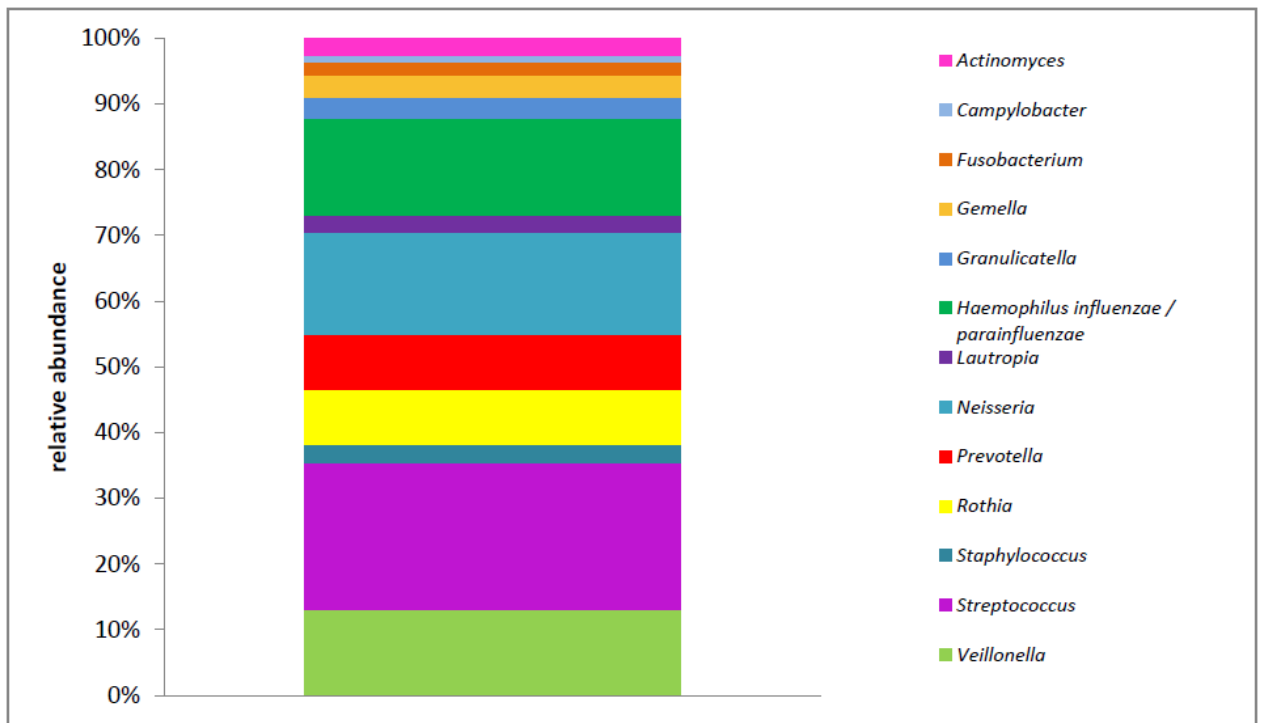

Supplement: Additional file 4: Fig. S1. — a - Number of core OTUs shared between samples at different prevalence thresholds. Only 9 OTUs were shared between all samples. Fig. S1b - Relative abundance of the most abundant taxa composing the common core microbiota (found across 50% of samples) present in the lungs of CF patients. Five phyla were found: Actinobacteria (10.4%), Bacteroidetes (9.6%), Firmicutes (43.7%), Fusobacteria (2.5%) and Proteobacteria (33.9%), and 13 predominant (i.e., relative abundance > 1%) genera: Haemophilus (14%), Campylobacter (1%), Neisseria (14.5%), Lautropia (2.4%), Fusobacterium (1.8%), Veillonella (12.3%), Staphylococcus aureus (2.6%), Streptococcus (2.1%), Granulicatella (2.9%), Gemella (3.3%), Prevotella (8.0%), Rothia (7.8%) and Actinomyces (2.4%). [file 40064_2015_1207_MOESM4_ESM.pdf]

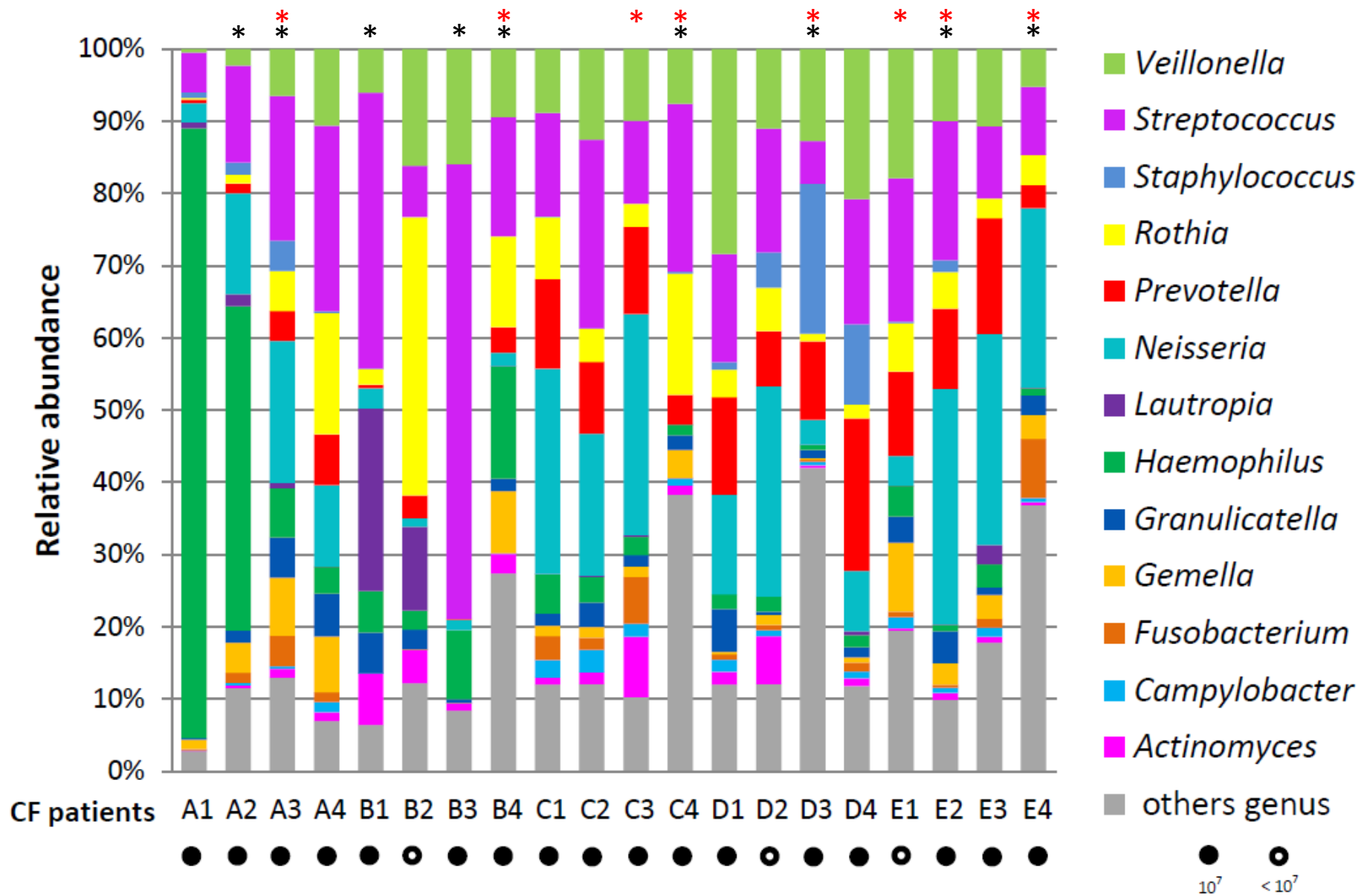

Supplement: Additional file 5: Fig. S2. — Bacterial community composition of serial samples obtained from 5 CF patients and quantification of total bacterial density. Relative abundance of each genus accounting for >1 % of the total bacterial community is shown and the relative abundance of other genera (accounting for <1 %) is shown in gray. Circles indicate total bacterial density (16S rRNA copies/mL sputum) based on quantitative PCR. Black stars and red stars indicate sputum samples positive for P. aeruginosa by culture and 454 pyrosequencing, respectively. [file 40064_2015_1207_MOESM5_ESM.pdf]
